# Trastuzumab deruxtecan *versus* chemotherapy for patients with HER2-low advanced breast cancer: A US-based cost-effectiveness analysis

**DOI:** 10.3389/fphar.2022.1025243

**Published:** 2022-10-28

**Authors:** Youwen Zhu, Kun Liu, Xiaolu Zhu, Qun Qin, Hong Zhu

**Affiliations:** ^1^ Department of Oncology, Xiangya Hospital, Central South University, Changsha, China; ^2^ National Clinical Research Center for Geriatric Disorders, Xiangya Hospital, Central South University, Changsha, China; ^3^ National Institution of Drug Clinical Trial, Xiangya Hospital, Central South University, Changsha, China

**Keywords:** HER2-low advanced breast cancer, trastuzumab-deruxtecan, cost-effectiveness analysis, quality-adjusted life years, Markov model

## Abstract

**Background:** In recent years, the rise of antibody–drug conjugates (ADCs) has changed the treatment paradigm for patients with HER2-low advanced breast cancer (ABC). DESTINY-Breast04 (NCT03734029) has demonstrated the antitumor activity of trastuzumab deruxtecan (T-DXd). However, the balance between the efficacy and cost of T-DXd remains undefined. Consequently, there is a great need to assess the cost-effectiveness of T-DXd for patients with HER2-low ABC when compared with chemotherapy.

**Methods:** A Markov decision-analytic model with a time horizon of 15 years was employed to estimate the costs and clinical efficacy of trials with the administration of T-DXd in contrast to chemotherapy alone as a later-line therapy in a group of patients with hormone receptor-positive (HR+) or negative (HR-) HER2-low ABC. The US payer perspective was taken into account when factors such as medical lifetime expenditure, incremental cost-effectiveness ratios (ICERs), and quality-adjusted life years (QALYs) were calculated. Sensitivity analyses were used to determine the model’s stability. A subgroup analysis was also conducted on the HR+/HER2-low cohort.

**Results:** T-DXd was associated with an improvement of 0.543, 0.558, and 0.789 QALYs when compared with treatment with chemotherapy for overall, HR+, and HR- HER2-low patients, respectively. However, incorporating T-DXd into later-line therapy led to increased costs ($161,406, $177,907, and $155,757), which causes the ICER for T-DXd to be $296,873, $318,944, and $197,355 per QALY. The cost of T-DXd and the patient’s weight were the most influential factors for ICER. T-DXd being the dominant strategy is about 1.5%, 0.5%, and 28.0% in overall, HR+, and HR- HER2-low ABC patients, respectively. In addition, the T-DXd regimen was not cost-effective in all subgroups.

**Conclusion:** Compared with chemotherapy, T-DXd was not cost-effective for patients with HER2-low ABC in the United States. However, it can provide more health benefits to patients with HR+/HER2-low ABC.

## Introduction

Breast cancer (BC) is one of the most common diseases and was the fifth most common cause of death across the globe in 2020, with a slightly increased incidence rate (by 0.5% annually) (Siegel et al., 2022; World Health Organization, 2022). In 2020, it is expected that there will be approximately 290,560 new cases diagnosed with BC in the United States, with 43,780 people dying from BC (Siegel et al., 2022). Among the women diagnosed with BC, 80–90% were diagnosed with an HER-2 (human epidermal growth factor receptor 2) negative tumor, which is characterized by a downregulated expression of the *HER2* gene (Slamon et al., 1987; Schettini et al., 2021). In HER2-negative tumors, hormone receptor-positive (HR+) and hormone receptor-negative (HR-) showcase substantial heterogeneity in terms of treatment sensitivity and prognosis, with a 0.8-fold difference in the 5-year relative survival (Burris et al., 2011; Fehrenbacher et al., 2020; Tarantino et al., 2020; National Cancer Institute, 2021; Schettini et al., 2021).

HER2-low expression has been defined as an immunohistochemical (IHC) assay score of 1+, or an IHC score of 2+ and a negative result *in situ* hybridization (ISH) (Fehrenbacher et al., 2020; Tarantino et al., 2020; Schettini et al., 2021). Although the NSABP B-47 (NCT01275677) study evaluated the efficacy of adjuvant chemotherapy with or without the monoclonal antibody trastuzumab in the treatment of subjects with HER2-low BC, the results were not satisfactory (Fehrenbacher et al., 2020). In particular, this study found that the addition of trastuzumab to adjuvant chemotherapy did not improve invasive disease-free survival (hazard ratio [HR], 0.98; 95% confidence interval (CI), 0.76 to 1.25; *p* = 0.85) (Fehrenbacher et al., 2020). For the moment, there is skepticism about the therapeutic prospect for patients with HER2-low because they cannot benefit from the traditional treatment of HER2, and therefore innovative treatment options must be developed.

Trastuzumab deruxtecan (T-DXd) is an ADC conjugated with anti-HER2 humanized monoclonal antibody (mAB) of tumor-associated antigen coupled with topoisomerase I inhibitor (DXd), which are connected by an enzyme-cleavable linker (Doi et al., 2017). With the improvement of T-DXd and its drug pharmaceutical properties, along with the increased bystander effect, clinicians have now turned their attention to T-DXd (Doi et al., 2017). The DESTINY-Breast04 (NCT03734029) phase III trial found that T-DXd treatment for patients with HER2-low ABC significantly improved the median overall survival (mOS, 23.4 *versus* 16.8 months; HR, 0.64; 95% CI, 0.49 to 0.84; *p* = 0.001) and progression-free survival (mPFS, 9.9 *versus* 5.1 months; HR, 0.50; 95% CI, 0.40 to 0.63; *p* = 0.003) when compared to chemotherapy (Modi et al., 2022). Surprisingly, T-DXd further showed significant antitumor activity for patients with HR+ (mOS, 23.9 months; mPFS, 10.1 months) or HR- (mOS, 18.2 months; mPFS, 8.5 months) HER2-low ABC (Modi et al., 2022). Based on these findings, T-DXd was included in the updated Guidelines of the National Comprehensive Cancer Network Clinical Practice (NCCN) as the preferred option for patients with HER2-low, who have received at least one prior line of chemotherapy for metastatic disease or if the tumor is HR+ and refractory to endocrine therapy in 2022(11). Consequently, T-DXd is changing the global landscape in the treatment of HER2-low ABC.

Although the T-DXd treatment is effective and safe for patients with HER2-low ABC, there is still a great need to assess the drug’s clinical benefit at a reasonable cost in light of the high price of recently approved novel drugs. Consequently, our investigation aims to evaluate the cost-effectiveness of T-DXd against chemotherapy as a later-line for treating HER2-low ABC and HR status from the economic perspective in the United States.

## Materials and methods

### Population and treatments

The patient cohort model in this inquiry was adapted from the DESTINY-Breast04 trial and involved 557 patients with HER2-low ABC. The study started on 27 December 2018 and lasted until 31 December 2021 (Modi et al., 2022). Of the 373 (67.0%) patients who were randomly assigned to the T-DXd group and the 184 (33.0%) patients who were assigned to the physician’s choice chemotherapy group, 331 (88.7%) and 163 (88.6%), respectively, comprised the HR+ cohort. In addition, the HR- cohort of patients comprised 42 (11.3%) and 21 (11.4%) individuals in the T-DXd and chemotherapy groups, respectively (Modi et al., 2022). The average age of the participants was 55 years, with a body weight of about 74 kg and a body surface area of 1.82 m^2^ (Table 1) (Le et al., 2016; Modi et al., 2022). All individuals with HER2-low ABC received at least first-line chemotherapy (Modi et al., 2022). In the T-DXd group, a dose of 5.4 mg T-DXd per kg of body weight was injected directly into the patient’s vein once every 3 weeks. Those patients that composed the physician’s choice chemotherapy group received anticancer medications such as eribulin (51.1%), capecitabine (20.1%), nab-paclitaxel (10.3%), gemcitabine (10.3%), or paclitaxel (8.2%) (Modi et al., 2022) at doses that complied to the Guidelines of NCCC (National Comprehensive Cancer Network Clinical, 2022). Detailed information on the dosage, method of administration, and price per unit of the drugs are provided in Supplementary Table S2 of Supplementary Material. Tumor measurements were performed every 6 weeks until the progression of the disease or the detection of unacceptable adverse events (AEs). In those two cases, the treatment was replaced with the best supportive care (BSC). In the T-DXd and chemotherapy group, 60 (16.2%) and 14 (8.1%) of the enrolled patients received BSC, respectively (Modi et al., 2022). Finally, every individual who had a treatment-related death received terminal care. This inquiry was guided according to the checklist of the reporting standards regarding the consolidated health economic evaluation (CHEERS) (Supplementary Material Supplementary Table S1).

**TABLE 1:** Model parameters: baseline values, ranges, and distributions for the sensitivity analysis.

Parameter	Baseline value	Range		References	Distribution
		Minimum	Maximum		
Weibull survival model for OS of chemotherapy					
Overall population	Scale = 0.011708, shape = 1.432,984	—	-	Modi et al. (2022)	-
Population with hormone receptor-positive	Scale = 0.011763, shape = 1.393,105	-	-	Modi et al. (2022)	-
Population with hormone receptor-negative	Scale = 0.050763, shape = 1.129,492	-	-	Modi et al. (2022)	-
Weibull survival model for PFS of chemotherapy					
Overall population	Scale = 0.164,483, shape = 0.903,477	-	-	Modi et al. (2022)	-
Population with hormone receptor-positive	Scale = 0.141,586, shape = 0.945,576	**-**	**-**	Modi et al. (2022)	**-**
Population with hormone receptor-negative	Scale = 0.25686, shape = 0.8906	-	-	Modi et al. (2022)	-
Weibull survival model for OS of trastuzumab deruxtecan					
Overall population	Scale = 0.007249, shape = 1.431,054	-	-	Modi et al. (2022)	-
Population with hormone receptor-positive	Scale = 0.004488, shape = 1.577,038	**-**	**-**	Modi et al. (2022)	**-**
Population with hormone receptor-negative	Scale = 0.026802, shape = 1.097747	-	-	Modi et al. (2022)	-
Weibull survival model for PFS of trastuzumab deruxtecan					
Overall population	Scale = 0.057066, shape = 1.074695	-	-	Modi et al. (2022)	-
Population with hormone receptor-positive	Scale = 0.049346, shape = 1.116,918	-	-	Modi et al. (2022)	-
Population with hormone receptor-negative	Scale = 0.11992, shape = 0.84977	-	-	Modi et al. (2022)	-
Rate of post-discontinuation therapy					
Chemotherapy group	0.081	0.065	0.097	Modi et al. (2022)	Beta
Trastuzumab deruxtecan group	0.162	0.130	0.194	Modi et al. (2022)	Beta
Risk for main AEs in the chemotherapy group					
Risk of neutropenia	0.407	0.326	0.488	Modi et al. (2022)	Beta
Risk of leukopenia	0.192	0.154	0.230	Modi et al. (2022)	Beta
Risk of increased aminotransferase levels	0.081	0.065	0.097	Modi et al. (2022)	Beta
Risk for main AEs in the trastuzumab deruxtecan group					
Risk of neutropenia	0.137	0.110	0.164	Modi et al. (2022)	Beta
Risk of anemia	0.081	0.065	0.097	Modi et al. (2022)	Beta
Risk of fatigue	0.075	0.060	0.090	Modi et al. (2022)	Beta
Risk of leukopenia	0.065	0.052	0.078	Modi et al. (2022)	Beta
Risk of thrombocytopenia	0.051	0.041	0.061	Modi et al. (2022)	Beta
Utility					
Utility PFS	0.700	0.560	0.840	Lloyd et al. (2006); Le et al. (2016)	Beta
Utility PD	0.500	0.400	0.600	Lloyd et al. (2006); Le et al. (2016)	Beta
Disutility					
Neutropenia	0.090	0.072	0.108	Ding et al. (2021)	Beta
Leukopenia	0.090	0.072	0.108	Ding et al. (2021)	Beta
Anemia	0.120	0.096	0.144	Le et al. (2016)	Beta
Thrombocytopenia	0.122	0.098	0.146	Le et al. (2016)	Beta
Fatigue	0.290	0.232	0.348	Liu et al. (2021)	Beta
Increased aminotransferase levels	0.308	0.246	0.370	Wang et al. (2021)	Beta
Drug cost, $/per cycle					
Chemotherapy	7,607	6,086	9,128	CMS (2022)	Gamma
Trastuzumab deruxtecan	20,114	16,091	24,137	CMS (2022)	Gamma
Cost of AEs, $					
Chemotherapy	7,870	6,296	9,444	Le et al. (2016); Wang et al. (2021); Zhu et al. (2021)	Gamma
Trastuzumab deruxtecan	2,585	2,068	3,102	Le et al. (2016); Wang et al. (2021); Zhu et al. (2021)	Gamma
Administration per cycle	352	282	422	Le et al. (2016)	Gamma
Follow-up per cycle	1,980	1,584	2,376	Zhang et al. (2019)	Gamma
Best supportive care per cycle	3,358	2,686	4,029	Wu et al. (2020)	Gamma
Immunohistochemical test per patient	123	98	148	Han et al. (2020)	Gamma
Terminal care per patient	2,844	2,275	3,413	Wang et al. (2021)	Gamma
Weight (kg)	74	59	89	Le et al. (2016)	Normal
Body surface area (meters^2^)	1.82	1.46	2.18	Le et al. (2016)	Normal
Discount rate	0.03	0	0.05	Ding et al. (2021)	Uniform

Abbreviation: OS, overall survival; PFS, progression-free survival; PD, disease progressed; AEs, adverse events.

### Model structure and transition probabilities

The three separate health states that established the 6-week cycle of the Markov model were PFS, PD, and death (Supplementary Material Supplementary Figure S1). This model was setup based on the combination of the efficacy of the treatment over time with the estimation of the transition probabilities. The latter was estimated from the DESTINY-Breast04 trial’s OS and PFS curves. The time-dependency transition probabilities in each Markov cycle were calculated based on the following formula: tp(tu) = 1 − exp{λ(t − u)γ − λtγ} (*λ* > 0, *?* > 0), where u is the Markov cycle and tu represents the arrival at state t after u Markov cycles. Over time, the patient’s health status deteriorated and led to mortality—more than 99% of the registered patients had died over the last 15 years. The Kaplan–Meier curves of the two groups were employed to select the points. The latter was combined with two criteria as the estimators of prediction error—that is, the Bayesian information criterion and the Akaike information criterion—to select the Weibull distribution that fitted the T-DXd and chemotherapy groups’ survival curve, respectively (Supplementary Material Supplementary Figure S2 and Supplementary eTable S4). Concerning the results from another study, we applied the Kaplan–Meier curves, while the shape and scale parameters for *γ* and *λ* distributions were calculated, respectively (Ding et al., 2021) (Table 1). The model was built with the TreeAge Software (TreeAge Pro 2021^®^, available at: https://www.treeage.com). The points were selected with the GetData Graph Digitizer (version 2.26, available at: http://www.getdata-graph-digitizer.com/index.php). R software (version 4.1.1, available at: http://www.rproject.org) was applied in the statistical data evaluation.

The model’s primary outcome was to calculate the overall costs, life years (LYs), quality-adjusted life years (QALYs), and incremental cost-effectiveness ratio (ICER). Based on published research, we determined the maximum price that the US payer is ready to pay for the corresponding therapy—in other words, the threshold of willingness-to-pay (WTP), which was $150,000/QALY (Ding et al., 2021). An annual discount rate of 3% on future medical costs and healthcare benefits was additionally implemented (Ding et al., 2021).

### Utility and cost

Health utility preference on a scale of 0 (death) to 1 (perfect health) was used in our analysis to reflect a particular health state, including PFS state, PD state, and death state. Because there were no reports regarding the health utility in the conducted clinical trials, the average health utility for PFS and disease progression statuses were assumed to be 0.70 and 0.50, respectively, which were taken from the published articles (Lloyd et al., 2006; Le et al., 2016). We have also corrected the mean health utility *via* the disutility values due to grade 3/4 AEs (Le et al., 2016; Ding et al., 2021; Liu et al., 2021; Wang et al., 2021) (Table 1).

We only examined direct expenditures, such as drugs, administration, IHC tests, follow-up patient, BSC, terminal care, and AEs (only included those with an incidence of grade 3/4 AEs in ≥5% of the cases) (Table 1). The prices of the drugs that were used were obtained from the official website for drug research (CMS, 2022). The remaining costs were derived come from published literature (Le et al., 2016; Wan et al., 2019; Zhang et al., 2019; Han et al., 2020; Wu et al., 2020; Liu et al., 2021; Wang et al., 2021; Zhu et al., 2021) (Table 1). According to the changes in prices paid by US consumers, the healthcare-related costs have been adjusted to the inflation rate in the United States for 2022 (US Bureau of Labor Statistics, 2022).

### Sensitivity analysis

The robustness of our conclusions was evaluated by a series of sensitivity analyses. We examined the value variation of 78 parameters in the employed model (ranging from −20% to 20%) to study the impact of examining individuals during a one-way sensitivity analysis on ICERs (Ding et al., 2021). To understand the employed model, 10000 Monte Carlo simulations were executed during the analysis of probability distribution. Every simulation randomly sampled the input model for the distribution. We have also taken the cost-effectiveness of the subgroup of patients with HR+/HER2-low ABC into consideration. Without reporting the survival curves of each group, the PFS curves of the T-DXd group were reconstructed from the overall PFS curves of the chemotherapy group and HR of each subgroup, as suggested by Ding et al. (2021).

## Results

### Cost-effectiveness results

T-DXd produced 1.869, 1.994, and 1.684 QALYs (3.275, 3.484, and 2.988 LYs) and chemotherapy gained 1.326, 1.436, and 0.895 QALYs (2.393, 2.598, and 1.626 LYs) for overall, HR+, and HR- HER2-low ABC patients, respectively. The cost of standard chemotherapy was calculated to be $119,970, $127,255, and $76,584, whereas for the T-DXd therapy it was estimated as $281,376, $305,162, and $232,341, respectively, for the aforementioned groups. For the T-DXd group, the ICERs cost was $296,873, $318,944, and $197,355 per QALY. Consequently, our results demonstrate that T-DXd was not the best strategy as a later-line therapy for both groups of patients with overall, HR+ and HR- HER2-low ABC in the US medical space, as illustrated in Table 2.

**TABLE 2:** Cost-effectiveness results.

Treatment	Total cost $	LYs	ICER $/LY^a^	QALYs	ICER $/QALY^b^
Overall population					
Chemotherapy	119,970	2.393	NA	1.326	NA
T-DXd	281,376	3.275	182,944	1.869	296,873
Population with hormone receptor-positive					
Chemotherapy	127,255	2.598	NA	1.436	NA
T-DXd	305,162	3.484	200,796	1.994	318,944
Population with hormone receptor-negative					
Chemotherapy	76,584	1.626	NA	0.895	NA
T-DXd	232,341	2.988	114,300	1.684	197,355

^a^ Compared to chemotherapy ($/LY).

^b^ Compared to chemotherapy ($/QALY).

Abbreviation: ICER, incremental cost-effectiveness ratio; LY, life year; QALY, quality-adjusted life year.

### Sensitivity analyses

**FIGURE 1:**
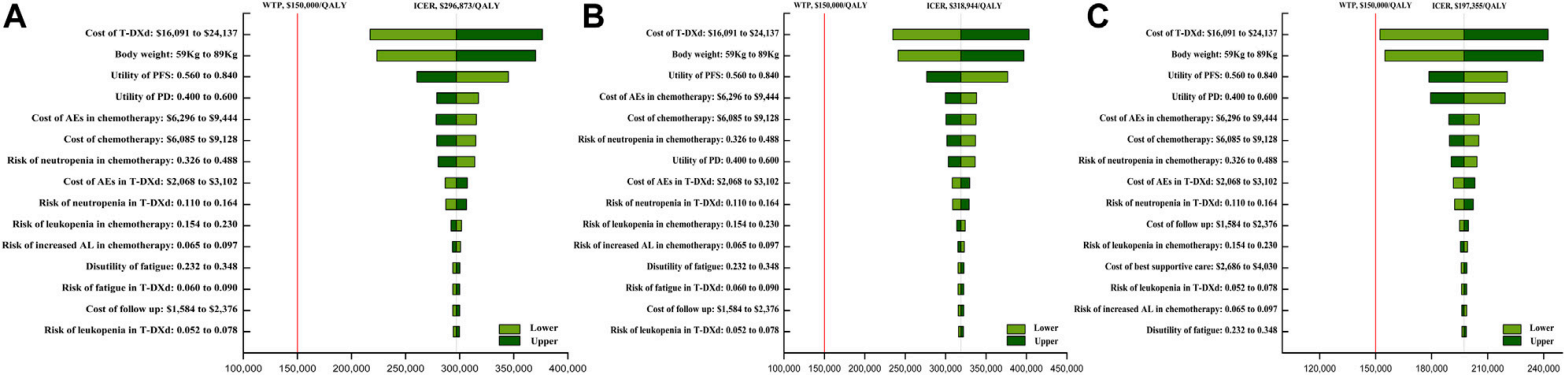
One-way sensitivity analyses of trastuzumab deruxtecan (T-DXd) strategy compared to chemotherapy strategy in the overall population **(A)**, population with hormone receptor-positive **(B)**, and population with hormone receptor-negative **(C)**. Abbreviation: ICER, incremental cost-effectiveness ratio; QALY, quality-adjusted life year; T-DXd, trastuzumab deruxtecan; AEs, adverse events; PFS, progression-free survival; PD, disease progressed; BSC, best supportive care; AL, aminotransferase levels.

The one-way sensitivity analysis revealed that the costs of T-DXd (varying from $16,091 to $24,137 each cycle, with the ICER ranging from $217,191/QALY to $376,576/QALY, $234,608/QALY to $403,302/QALY, and $152,336/QALY to $242,385/QALY in overall, HR+, and HR- HER2-low ABC patients, respectively), body weight (varying from 59 kg to 89 kg, with the ICER ranging from $223,519/QALY to $370,227/QALY, $241,113/QALY to $396,774/QALY, and $154,971/QALY to $239,739/QALY in overall, HR+, and HR- HER2-low ABC patients, respectively), the costs of chemotherapy, the costs of AEs in chemotherapy, and the utility of PFS had a significant impact on the model (Figure 1). In addition, the cost of the IHC test and the cost of terminal care had a small impact on the model.

**FIGURE 2:**
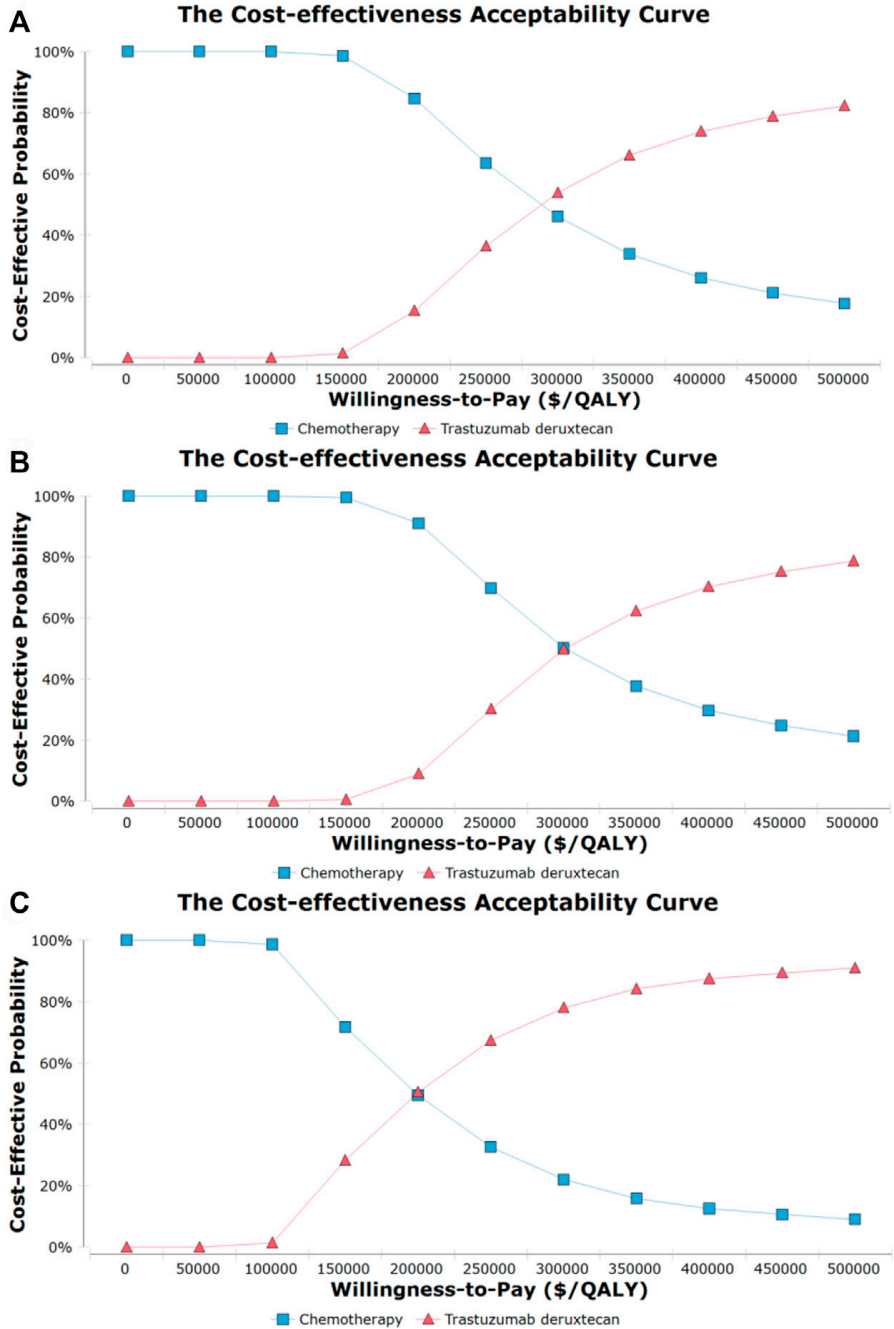
Cost-effectiveness acceptability curves for trastuzumab deruxtecan (T-DXd) strategy compared to chemotherapy strategy in the overall population **(A)**, population with hormone receptor-positive **(B)**, and population with hormone receptor-negative **(C)**. Abbreviation: QALY, quality-adjusted life year.

The probability sensitivity analysis using the cost-effectiveness acceptability curve (Figure 2) and scatter plot (Supplementary Figure S3) revealed that the probability of T-DXd being the dominant strategy is about 1.5%, 0.5%, and 28.0% in overall, HR+, and HR- HER2-low ABC patients, respectively, at the WTP of 150,000/QALY. Furthermore, we found that the benefits at the relevant price of T-DXd treatment changed with the fluctuation of WTP. For example, on the occasion of a two times rise in the threshold of the WTP, namely, 300 000$ per QALY, the T-DXd had a 50% probability to be cost-effective when compared with chemotherapy.

Interestingly, the T-DXd treatment proved beneficial in decreasing the chance of death in most subgroups. Moreover, the ICERs of the -DXd *vs.* chemotherapy ranged from $193,455/QALY to $486,154/QALY. The probability sensitivity analysis indicated that T-DXd was cost-effective with probabilities ranging between 0% and 35.5% (Supplementary eTable S3).

## Discussion

BC has become one of the highest-priced malignant tumors worldwide (Sullivan et al., 2011). The cost of treating women with ABC reached 75.4 billion US dollars in 2020 and has since increased by 4.3%. This puts BC into the category of cancers with the largest increase in healthcare costs (Tartari et al., 2017; Gogate et al., 2021). Currently, patients with HER2-low ABC have limited treatment options after progression during primary therapy. Among them, the available targeted therapies appeared costly with less successful clinical outcomes for these patients (Burris et al., 2011; Cortes et al., 2011; Kaufman et al., 2015; Fehrenbacher et al., 2020; Cook et al., 2021). The development of T-DXd novel drugs has shown great potential in the field of HER2-low expression and has attracted widespread attention. Subsequently, the analysis of the cost-effectiveness of T-DXd has proven to be necessary when the clinical practice guidelines suggest its broad application.

To date, there is no evidence on the assessment of the cost-efficacy of T-DXd in treating subjects with HER2-low BC diagnosis. Only a few studies have been published discussing the cost-effectiveness of trastuzumab emtansin (T-DM1), mostly for the therapeutic purposes of individuals struggling with HER2-positive ABC. Several reports have evaluated T-DM1 as a second-approach therapy in contrast with combined chemotherapy from a payer’s viewpoint in countries such as the United States, China, United Kingdom, and Spain. These studies reached a consensus that T-DM1 was not a beneficial strategy for the treatment of HER-low BC at a relevant price, probably due to the high price of ADCs (Miranda Romero and Marín Gil, 2015; Le et al., 2016; Squires et al., 2016; Zhang et al., 2021). However, one report confirmed the greater cost-effectiveness of T-DM1 in comparison with chemotherapy alone in the United States (Le et al., 2016). These studies shed light on the possible differences in the cost-effectiveness of ADCs retrieved from the analyses for different payers with the same treatment regimen. The reason for these differences may be that local affordability and market assessment programs differ. Therefore, when an approved drug is widely used in clinical practice, it is equally important for its cost-effectiveness to be proven in different regions.

To our knowledge, this study is the first to build a 15-year Markov model as an instrument to contrast the cost-effectiveness of T-DXd with chemotherapy as later-line treatment for patients with HER2-low ABC from a US payer’s perspective. Our study shows that the employment of T-DXd in comparison with physician’s choice chemotherapy produced 0.543 QALYs that increased by $161,406, thus leading to an ICER of $296,873/QALY, which was significantly higher than the WTP standard of $150,000/QALY in the United States. The additional costs associated with T-DXd mainly represented the drug price. Therefore, T-DXd was not a dominant strategy from the point of view of US payers. This means that the high prices of innovative drugs widely used in clinical practice are the main problem. Further analysis has shown that T-DXd cost had a pivotal role in the one-sensitivity analysis. T-DXd therapy was considered to be a cost-effective strategy in the case of a more than 40% decrease in the T-DXd price or in the case of a more than 2.5 times increase in the price of chemotherapy. Therefore, considerable price adjustments are required to enable a wider range of acceptable ICERs. Although the cost for specific indications has the potential to maximize the revenue and decrease the buyer’s excess benefit, there is an agreement among researchers that the prices of medicines and their potential medical usefulness seem to have no or little correlation to each other (Mailankody and Prasad, 2015; Chandra and Garthwaite, 2017). Subsequently, it is necessary to overcome the administrative challenges in the United States by linking the costs and efficacy of the drugs, and encouraging the development of therapies with high impact. Body weight was another important factor in our study. Surprisingly, the cost-effectiveness of T-DXd was low at the WTP inception at 150 000$/QALY for patients weighing more than 43 kg. Nevertheless, most of the enrolled patients weighed more than 40 kg (Darnis et al., 2012; da Silva et al., 2017), which raised an ethical issue of debiting emaciated individuals less for the same amount of money for a life-prolonging procedure. The potential reasons for this might be the adjustment of the T-DXd dose to the patient’s body weight and the number of disposable vials rather than the administered dose when the drug cost was calculated. Heavier patients required more T-DXd, which increased the financial burden. Our recommendation in this case is to arrange the patient’s medication bottles on the same day. However, there are some safety concerns about sharing the vials and the US Centers for Disease Control and Prevention claim that each patient should use their vial for single usage (Centers for Disease Control and Prevention, 2022).

Our findings from the executed analysis demonstrate a lack of price-efficacy of the T-DXd in patients with HR+ or HR- HER2-low ABC, with detected ICERs of about $318,944/QALY and $197,355/QALY, respectively. Even though it was not cost-effective, T-DXd provided greater health benefits for patients with HR+/HER2-low ABC. This is consistent with the findings of several previously published studies (Gampenrieder et al., 2021; Horisawa et al., 2022). A recent retrospective study involved 4,977 Japanese patients for comparison of the prognosis of BC disease concerning the HR status among patients with HER2-low BC (Horisawa et al., 2022). The authors found that the HR-/HER2-low cases had a worse prognosis than the HR+/HER2-low cases, with 5-year OS (96.7% and 86.5%, respectively) and 5-year PFS (91.6% and 78.7%, respectively). Another retrospective study that included 1,973 Austrian patients showed that individuals struggling with HR+/HER2-low and HR-/HER2-low metastatic BC had higher 5-year OS (11% and 33%, respectively) and 5-year PFS (37% and 6%, respectively) (Gampenrieder et al., 2021). Due to the high cost of new HER2-ADC drugs, therapeutic strategies for treating patients with HER2-low breast cancer should be considered in the context of HR status in the context of cost-effectiveness and optimal choice, and early testing of such prognostic factors is critical.

As with most cost-effectiveness analyses, our study has observed some limitations. First, we acknowledge that phase III DESTINY-Breast04 is the only trial that randomly compares T-DXd cost-effectiveness with chemotherapy in individuals struggling with HER2-low ABC. This trial is characterized by its large scale and proper plan, However, the model depends on the trial results, which means that any bias in the test will have a serious impact on the outcome of this study. Second, the extended benefit of T-DXd in the current model was inferred from the data retrieved of the Destiny-BREAST04 trial, which was exposed to ambiguity. To assess the ambiguity, we performed a series of sensitivity and subgroup analyses. However, the prolonged benefits of T-DXd remained unclear. Therefore, more data are needed to validate the model *versus* the prolonged survival data. Third, due to the shortage of subgroup survival data and curves for HR+/HER2-low, as well as the reduction of the strength of the results because of the small size of the samples, we have carefully interpreted the results of the subsection analysis. Fourth, due to sparse data on utility values, we have used such values from the published literature. While this estimate cannot be regarded as ideal, we have executed analyses that included utility value variability. Finally, we adjusted the mean health utility using disutility values of AEs but we have only considered disutility with an incidence of grade 3 or higher AEs in ≥5%, which led to the overstatement or understatement of the utility values. However, the conducted analysis showcased the small influence of the disutility of AEs on economic outcomes.

## Conclusion

This study has revealed that the widespread use of innovative drugs requires the drug price and drug dosage to be balanced for the most cost-effective treatment to be obtained. From a US payer’s perspective, our study showed that T-DXd was not cost-effective for patients with HER2-low ABC. Furthermore, we have provided evidence that the HR status should be taken into consideration in the price-efficacy evaluation because T-DXd provides additional health benefits for patients with HR+/HER-low ABC.

## Data Availability

The original contributions presented in the study are included in the article/Supplementary Material; further inquiries can be directed to the corresponding author.
