# Supplementary material for: Trastuzumab deruxtecan versus chemotherapy for patients with HER2-low advanced breast cancer: A US-based cost-effectiveness analysis
Source: Front Pharmacol. 2022 Oct 28;13:1025243. doi: 10.3389/fphar.2022.1025243 (PMC9650214; doi:10.3389/fphar.2022.1025243)
Supplement: Supplementary file 1 [file DataSheet1.PDF]

eFigure 1. Model structure.

eFigure 2. Kaplan-Meier Curve Fitting and Extrapolation.

eFigure 3. Probability Sensitivity Analysis Scatter Plot.

eTable 1. CHEERS Checklist.

eTable 2. Drug dose and cost.

eTable 3. Results of subgroup analyses.

eTable 4. Summary of statistical goodness-of-fit of K-M curve.

eFigure 1. Model Structure.

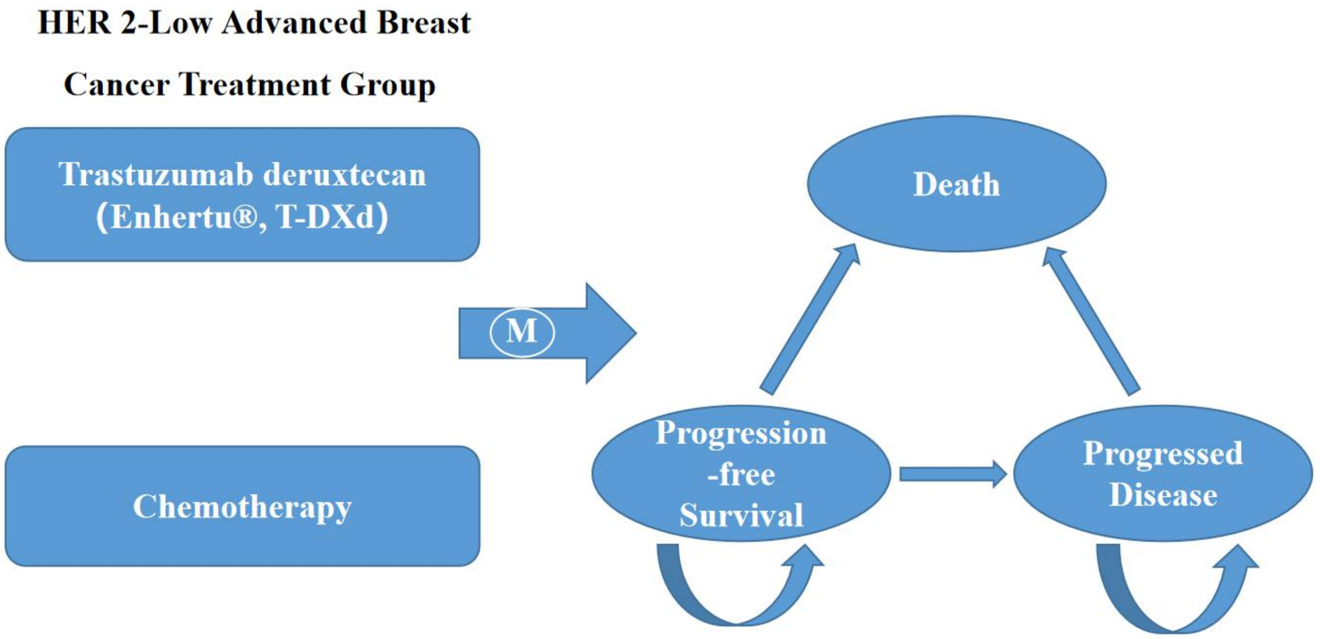

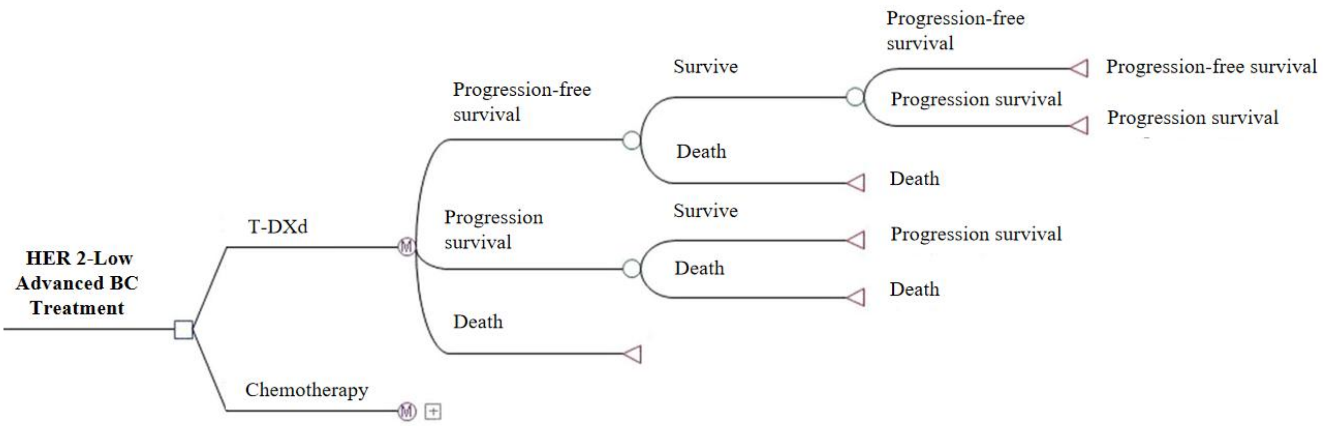

Abbreviation: M, Markov; T-DXd, trastuzumab deruxtecan; BC, metastatic breast cancer.

eFigure 2. Kaplan-Meier Curve Fitting and Extrapolation.

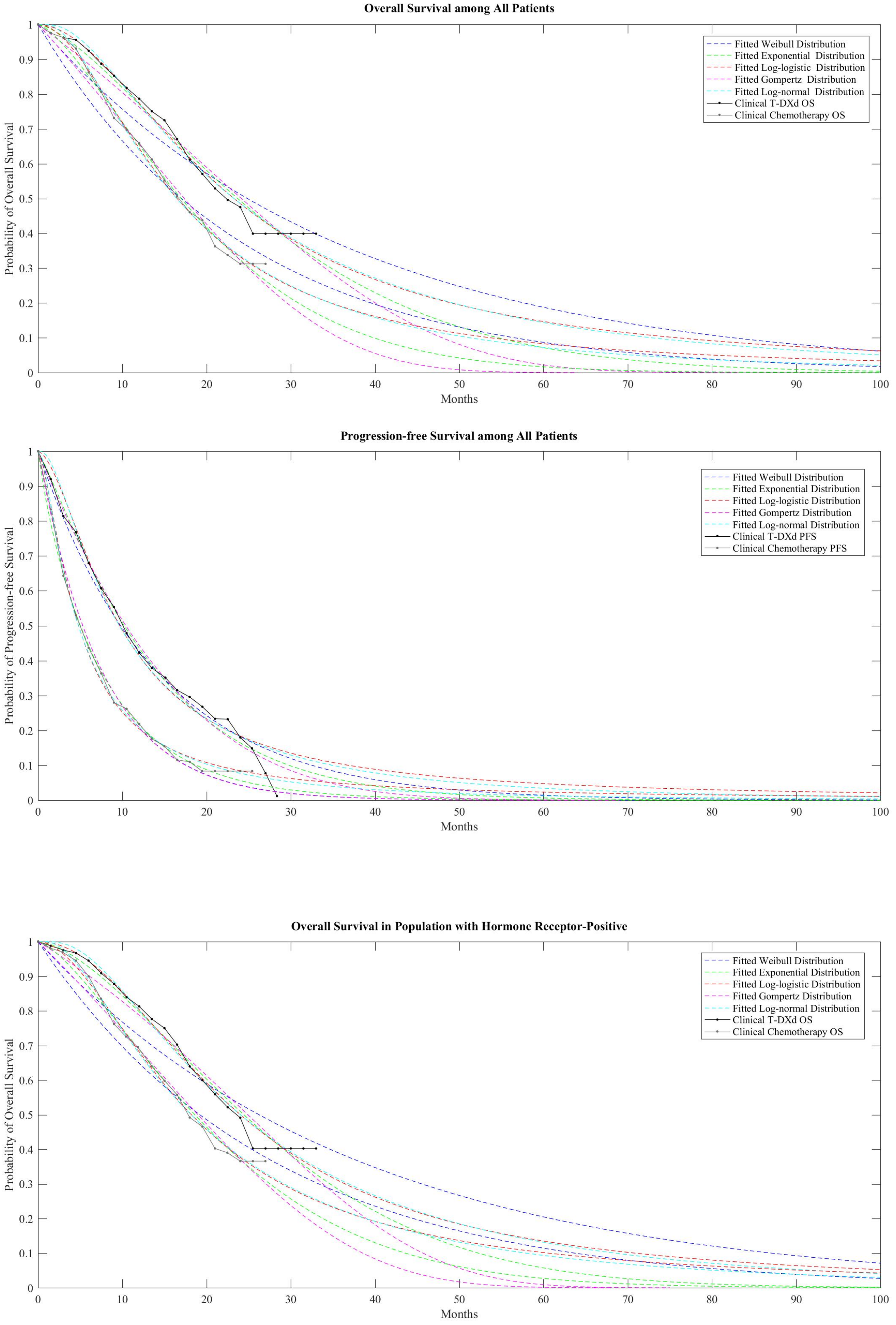

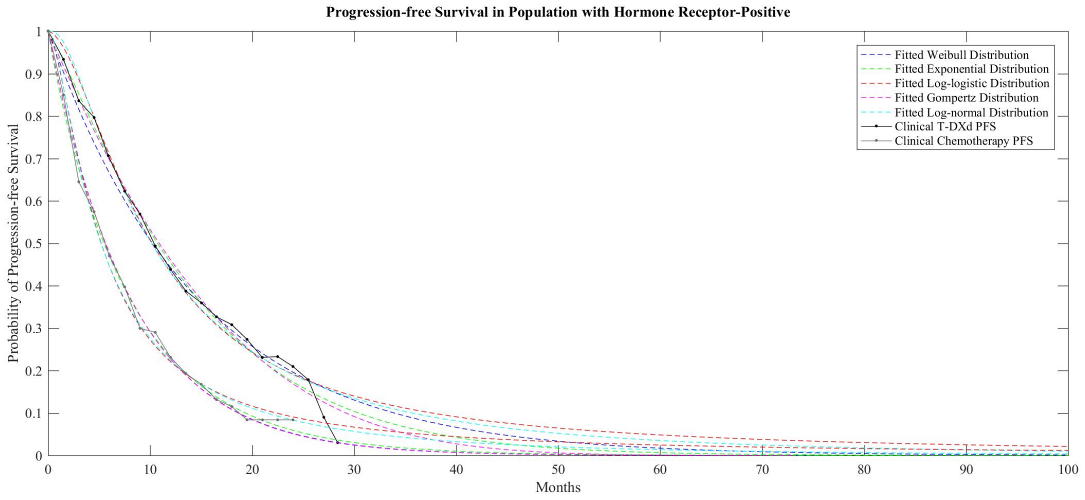

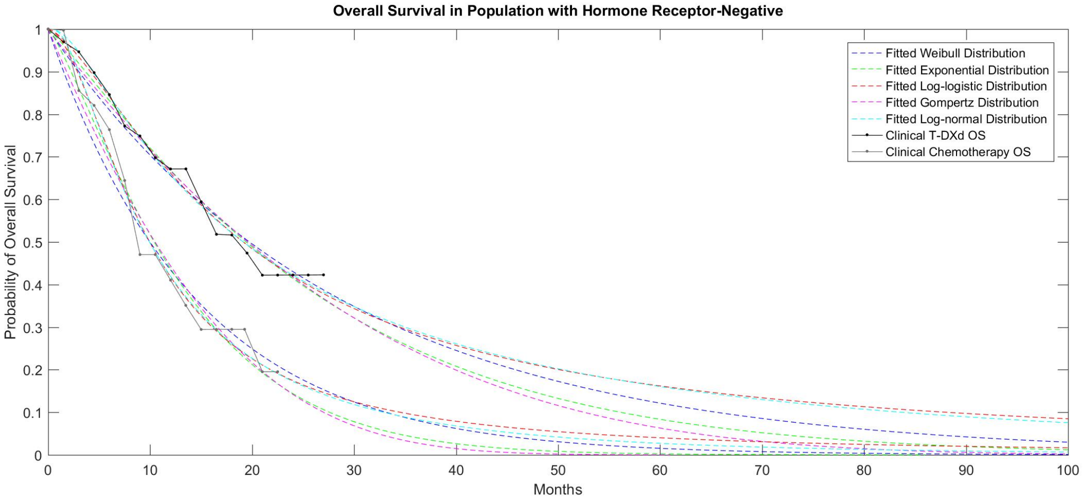

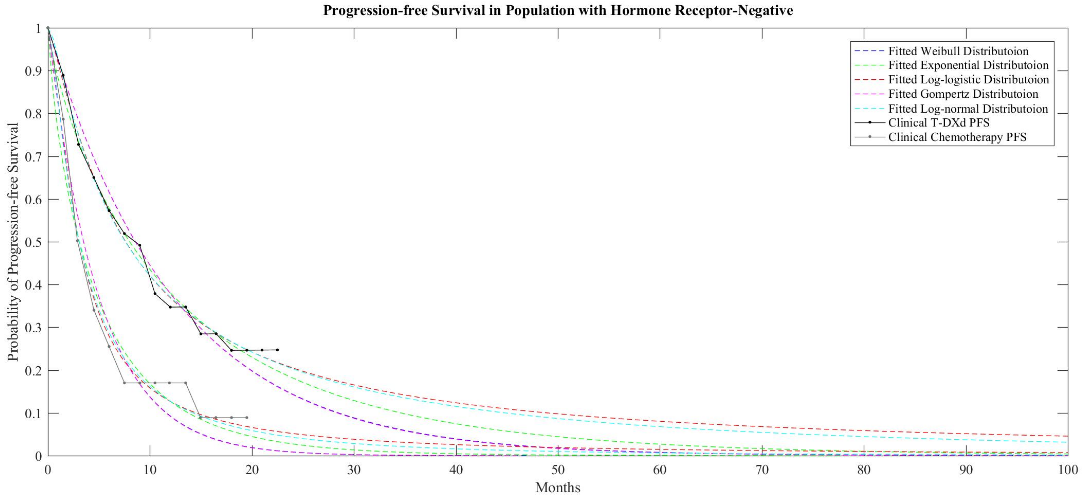

Abbreviation: T-DXd, trastuzumab deruxtecan; OS, overall survival; PFS, progression-free survival.

eFigure 3. Probability Sensitivity Analysis Scatter Plot.

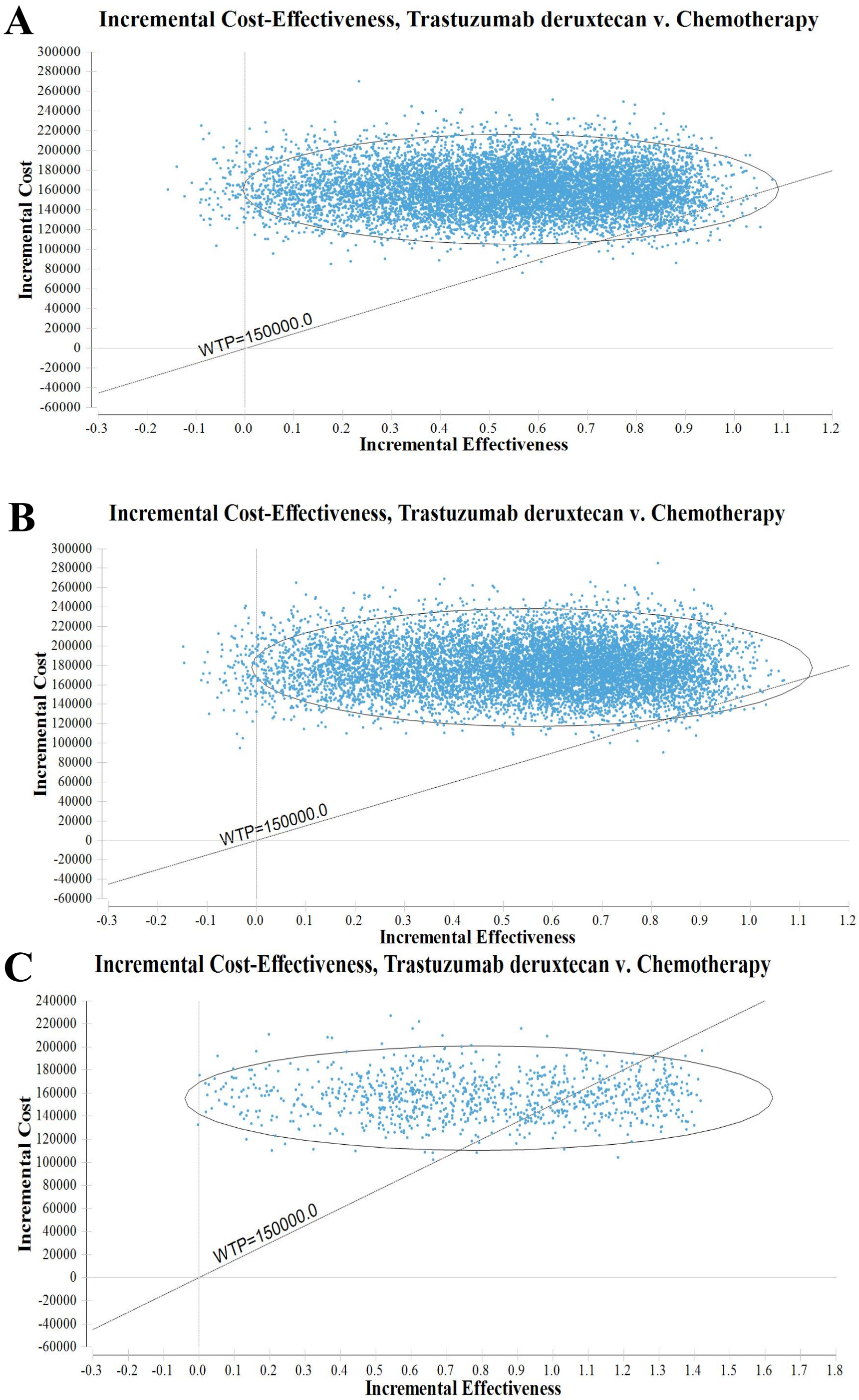

Abbreviation: WTP, willingness-to-pay.

Each point in the diagram represents a simulation result of 10,000 Monte Carlo simulation. Ellipse represent the 95% CI and dotted line represent WTP of $150,000/QALY in the overall population (A), population with hormone receptor-positive (B), and population with hormone receptor-negative (C) respectively. Points to the right of the dotted line are considered cost-effective.

eTable 1. CHEERS Checklist.

Section/item	Item No	Recommendation	Reported on page No
**Title and abstract**			
Title	1	Identify the study as an economic evaluation or use more specific terms such as “cost-effectiveness analysis”, and describe the interventions compared.	I
Abstract	2	Provide a structured summary of objectives, perspective, setting, methods (including study design and inputs), results (including base case and uncertainty analyses), and conclusions.	II
**Introduction**			
Background and objectives	3	Provide an explicit statement of the broader context for the study.	1, 2
		Present the study question and its relevance for health policy or practice decisions.	
**Methods**			
Target population and subgroups	4	Describe characteristics of the base case population and subgroups analysed, including why they were chosen.	2, 3, 5
Setting and location	5	State relevant aspects of the system(s) in which the decision(s) need(s) to be made.	3, 4
Study perspective	6	Describe the perspective of the study and relate this to the costs being evaluated.	4, 5
Comparators	7	Describe the interventions or strategies being compared and state why they were chosen.	2, 3
Time horizon	8	State the time horizon(s) over which costs and consequences are being evaluated and say why appropriate.	3
Discount rate	9	Report the choice of discount rate(s) used for costs and outcomes and say why appropriate.	4
Choice of health outcomes	10	Describe what outcomes were used as the measure(s) of benefit in the evaluation and their relevance for the type of analysis performed.	4
Measurement of effectiveness	11a	*Single study-based estimates:* Describe fully the design features of the single effectiveness study and why the single study was a sufficient source of clinical effectiveness data.	3, 4

	11b	*Synthesis-based estimates:* Describe fully the methods used for identification of included studies and synthesis of clinical effectiveness data.	
Measurement and valuation of preference based outcomes	12	If applicable, describe the population and methods used to elicit preferences for outcomes.	Not applicable
Estimating resources and costs	13a	*Single study-based economic evaluation:* Describe approaches used to estimate resource use associated with the alternative interventions. Describe primary or secondary research methods for valuing each resource item in terms of its unit cost. Describe any adjustments made to approximate to opportunity costs.	2-5
	13b	*Model-based economic evaluation:* Describe approaches and data sources used to estimate resource use associated with model health states. Describe primary or secondary research methods for valuing each resource item in terms of its unit cost. Describe any adjustments made to approximate to opportunity costs.	
Currency, price date, and conversion	14	Report the dates of the estimated resource quantities and unit costs. Describe methods for adjusting estimated unit costs to the year of reported costs if necessary. Describe methods for converting costs into a common currency base and the exchange rate.	4, 5
Choice of model	15	Describe and give reasons for the specific type of decision-analytical model used. Providing a figure to show model structure is strongly recommended.	3<br>eFigure 1
Assumptions	16	Describe all structural or other assumptions underpinning the decision-analytical model.	2-5
Analytical methods	17	Describe all analytical methods supporting the evaluation. This could include methods for dealing with skewed, missing, or censored data; extrapolation methods; methods for pooling data; approaches to validate or make adjustments (such as half cycle corrections) to a model; and methods for handling population heterogeneity and uncertainty.	3, 4<br>eFigure 2<br>eTable 5
**Results**			

Study parameters	18	Report the values, ranges, references, and, if used, probability distributions for all parameters. Report reasons or sources for distributions used to represent uncertainty where appropriate. Providing a table to show the input values is strongly recommended.	2-5<br>Table 1
Incremental costs and outcomes	19	For each intervention, report mean values for the main categories of estimated costs and outcomes of interest, as well as mean differences between the comparator groups. If applicable, report incremental cost-effectiveness ratios.	6<br>Table 2
Characterising uncertainty	20a	*Single study-based economic evaluation:* Describe the effects of sampling uncertainty for the estimated incremental cost and incremental effectiveness parameters, together with the impact of methodological assumptions (such as discount rate, study perspective).	6
	20b	*Model-based economic evaluation:* Describe the effects on the results of uncertainty for all input parameters, and uncertainty related to the structure of the model and assumptions.	
Characterising heterogeneity	21	If applicable, report differences in costs, outcomes, or cost-effectiveness that can be explained by variations between subgroups of patients with different baseline characteristics or other observed variability in effects that are not reducible by more information.	Not applicable
**Discussion**			
Study findings, limitations, generalisability, and current knowledge	22	Summarise key study findings and describe how they support the conclusions reached. Discuss limitations and the generalisability of the findings and how the findings fit with current knowledge.	6-10
**Other**			
Source of funding	23	Describe how the study was funded and the role of the funder in the identification, design, conduct, and reporting of the analysis. Describe other non-monetary sources of support.	11
Conflicts of interest	24	Describe any potential for conflict of interest of study contributors in accordance with journal policy. In the absence of a journal policy, we recommend authors comply with International Committee of Medical Journal Editors recommendations.	11

A good template page for CHEERS Checklist is as follows: http://www.ispor.org/TaskForces/EconomicPubGuidelines.asp.

Reference:

Husereau D, Drummond M, Petrou S, et al. Consolidated health economic evaluation reporting standards (CHEERS) —Explanation and elaboration: A report of the ISPOR health economic evaluations publication guidelines good reporting practices task force. Value Health 2013;16:231-50.

eTable 2. Drug dose and cost.

Drug	Dose	Time	Unit costs ($/mg)
Trastuzumab deruxtecan (Enhertu®, T-DXd)	5.4 mg/kg	Administered intravenously every 3 weeks	25.167
Eribulin	1.23 mg/m^2, on days 1 and 8	Administered intravenously every 3 weeks	1,328.030
Capecitabine	1250 mg/m^2, on days 1 to 14	Administered orally twice per day every 3 weeks every 3 weeks	0.003
Nab-paclitaxel	260 mg/m^2, on day 1	Administered intravenously every 3 weeks	14.693
Gemcitabine	1000 mg/m^2, on days 1 and 8	Administered intravenously every 3 weeks	0.019
Paclitaxel	175 mg/m^2, on day 1	Administered intravenously every 3 weeks	0.124

eTable 3. Results of subgroup analyses.

Subgroup	No. of patients		PFS HR (95% CI)	ICER, $/QALY	Cost-effectiveness probability of T-DXd at WTP, %
	T-DXd	Chemo			$150,000/QALY
**Prior CKD4/6 inhibitor**					
Yes	149	74	0.55 (0.42 to 0.73)	300,135	1.0
No	60	35	0.42 (0.28 to 0.64)	318,944	0
**IHC status**					
IHC 1+	119	66	0.48 (0.35 to 0.65)	365,391	0
IHC 2+/ISH-	92	44	0.55 (0.38 to 0.80)	333,764	0
**Prior lines of chemotherapy in the metastatic setting**					
1	129	63	0.54 (0.40 to 0.73)	304,739	0.9
≥ 2	81	47	0.47 (0.33 to 0.68)	338,847	0.3

**Age, years**					
＜ 65	170	79	0.51 (0.39 to 0.67)	318,944	0.5
≥ 65	41	31	0.47 (0.29 to 0.77)	338,847	0.3
**Race**					
White	100	43	0.64 (0.44 to 0.91)	261,440	6.5
Asian	83	54	0.40 (0.28 to 0.56)	376,561	0
Other	25	11	0.83 (0.41 to 1.69)	193,455	35.5
**ECOG performance status**					
0	116	55	0.56 (0.40 to 0.77)	295,596	1.8
1	95	55	0.45 (0.32 to 0.64)	349,245	0.1
**Visceral disease at baseline**					
Yes	196	100	0.54 (0.42 to 0.69)	304,739	0.9
No	15	10	0.23 (0.09 to 0.55)	486,154	0

Abbreviation: T-DXd, trastuzumab deruxtecan; Chemo, chemotherapy; PFS HR, progression-free survival hazard ratio; CI, confidence interval;

QALYs, quality-adjusted life-years; ICER, incremental cost-effectiveness ratio; WTP, Willingness-to-pay; CDK4/6, cyclin-dependent kinases 4 and 6; IHC, immunohistochemical; ISH, in situ hybridization; ECOG, the Eastern Cooperative Oncology Group.

eTable 4. Summary of statistical goodness-of-fit of K-M curve.

	Exponential	Weibull	Gompertz	Log-logistic	Log-normal
**Overall population**					
**T-DXd OS curve**					
AIC	25.2760	23.1728	25.4148	25.1288	25.0106
BIC	27.5470	24.3083	27.6858	27.3998	27.2816
**Chemotherapy OS curve**					
AIC	25.2659	22.8856	25.4024	25.0445	24.9640
BIC	27.1547	23.8301	27.2913	26.9334	26.8529
**T-DXd PFS curve**					
AIC	45.3949	42.3351	45.8738	44.1059	44.2430
BIC	47.3864	43.3308	47.8653	46.0974	46.2345
**Chemotherapy PFS curve**					
AIC	62.0850	61.1453	64.2744	57.9697	58.6173
BIC	62.9754	62.9260	66.0552	59.7505	60.3981
**Population with hormone receptor-positive**					
**T-DXd OS curve**					
AIC	24.1597	22.0578	24.3375	24.0118	23.8888
BIC	26.4307	23.1933	26.6085	26.2828	26.1598
**Chemotherapy OS curve**					
AIC	22.6537	20.4920	22.7590	22.5220	22.4371
BIC	24.5426	21.4364	24.6479	24.4109	24.3260
**T-DXd PFS curve**					

AIC	43.8601	40.6656	44.2024	42.7183	42.8098
BIC	45.8516	41.6613	46.1939	44.7098	44.8013
**Chemotherapy PFS curve**					
AIC	53.2185	52.3296	54.4375	50.7285	51.1961
BIC	54.8849	53.1628	56.1039	52.3949	52.8626
**Population with hormone receptor-negative**					
**T-DXd OS curve**					
AIC	22.0184	20.0199	22.0621	21.9385	21.8721
BIC	23.9073	20.9643	23.9510	23.8274	23.7610
**Chemotherapy OS curve**					
AIC	29.8558	26.9856	29.7464	29.5103	29.4251
BIC	31.4010	27.7582	31.2916	31.0555	30.9703
**T-DXd PFS curve**					
AIC	32.2522	31.1136	33.1394	32.0113	31.9972
BIC	33.7974	31.8862	34.6846	33.5565	33.5424
**Chemotherapy PFS curve**					
AIC	56.0474	50.8640	58.2714	49.4359	49.8114
BIC	56.6864	52.1421	59.5496	50.7140	51.0895

Abbreviation: T-DXd, trastuzumab deruxtecan; T-DM1, trastuzumab emtansine; OS, overall survival; PFS, progression-free survival; AIC, Akaike's information criterion; BIC, Bayesian information criterion.

According to the AIC and BIC value of the distributions and the visual fitting of the curves (eFigure 2) showed that the Weibull distribution is probably the most reasonable parametric survival model. Weibull distribution is flexible and widely used for matching patients with three states over time, because it can monotonously increase or decrease risk functions, and it is suitable for estimating events occurring during early

follow-up work.
